# Insulin- and Warts-Dependent Regulation of Tracheal Plasticity Modulates Systemic Larval Growth during Hypoxia in *Drosophila melanogaster*

**DOI:** 10.1371/journal.pone.0115297

**Published:** 2014-12-26

**Authors:** Daniel M. Wong, Zhouyang Shen, Kristin E. Owyang, Julian A. Martinez-Agosto

**Affiliations:** 1 Department of Human Genetics, David Geffen School of Medicine, University of California Los Angeles, Los Angeles, California, United States of America; 2 Department of Pediatrics, David Geffen School of Medicine, University of California Los Angeles, Los Angeles, California, United States of America; 3 Molecular Biology Institute, University of California Los Angeles, Los Angeles, California, United States of America; 4 Jonsson Comprehensive Cancer Center, University of California Los Angeles, Los Angeles, California, United States of America; Institut de Génomique Fonctionnelle, France

## Abstract

Adaptation to dynamic environmental cues during organismal development requires coordination of tissue growth with available resources. More specifically, the effects of oxygen availability on body size have been well-documented, but the mechanisms through which hypoxia restricts systemic growth have not been fully elucidated. Here, we characterize the larval growth and metabolic defects in Drosophila that result from hypoxia. Hypoxic conditions reduced fat body opacity and increased lipid droplet accumulation in this tissue, without eliciting lipid aggregation in hepatocyte-like cells called oenocytes. Additionally, hypoxia increased the retention of Dilp2 in the insulin-producing cells of the larval brain, associated with a reduction of insulin signaling in peripheral tissues. Overexpression of the wildtype form of the insulin receptor ubiquitously and in the larval trachea rendered larvae resistant to hypoxia-induced growth restriction. Furthermore, Warts downregulation in the trachea was similar to increased insulin receptor signaling during oxygen deprivation, which both rescued hypoxia-induced growth restriction, inhibition of tracheal molting, and developmental delay. Insulin signaling and loss of Warts function increased tracheal growth and augmented tracheal plasticity under hypoxic conditions, enhancing oxygen delivery during periods of oxygen deprivation. Our findings demonstrate a mechanism that coordinates oxygen availability with systemic growth in which hypoxia-induced reduction of insulin receptor signaling decreases plasticity of the larval trachea that is required for the maintenance of systemic growth during times of limiting oxygen availability.

## Introduction

The regulation of organismal growth requires the integration of cell, tissue, and systemic signals that determine the final body size of a developing organism. Furthermore, a response to environmental stimuli is required for the adaptation of growth to available resources. However, the mechanisms through which organismal size is coordinated with environmental cues remain poorly understood. Specifically, hypoxia, or low oxygen availability, has been shown to restrict larval and adult size [1]–[5] in Drosophila, but the mechanism responsible for this growth restriction has not been fully elucidated.

In Drosophila, growth mainly occurs during the three larval growth transitions, or instars, that are regulated by ecdysone pulses [6]. Once final larval size is attained, body size no longer increases during the adult stage [7]. During this larval period, there is significant growth of imaginal and polyploid tissues [7], [8], which is needed to support tissue remodeling during metamorphosis. Upon attaining critical weight, defined as the minimum larval mass at which further growth is no longer required for normal progression towards pupariation [9], larvae commit to entering metamorphosis [6], a point at which final body size becomes determined. Attainment of critical weight is dependent on proper metabolic regulation, as defects in energy homeostasis have been associated with larval growth restriction [10]–[13]. Several studies in Drosophila have demonstrated that systemic growth is controlled through specific genetic programs found in and environmental responses mounted by distinct tissues and cell types [14], [15], including brain [16], [17], fat body [18]–[22], oenocytes [23], ring gland [19], [24]–[27], muscle [28], and gut [29]. However, of all these tissues, only the prothoracic gland [5] has been shown to coordinate organismal size with oxygen availability.

The Drosophila insulin signaling system plays a crucial role in regulating systemic growth [14], [15], [30]. There are eight Drosophila insulin-like peptides (Dilps) that function as ligands for the single Drosophila insulin receptor in target tissues [31]. Previous studies examining the function of the insulin-producing cells (IPCs) of the larval brain, which produce and secrete Dilp1, Dilp2, Dilp3, and Dilp5, have demonstrated the critical role that Dilps play as systemic growth hormones [16], [20], [22], [31]. In larval physiology, the rate at which growth occurs is regulated by insulin signaling [6]. Growth rate is tightly coupled with the duration of the growth period, which is determined by ecdysone production in the larval prothoracic gland [6]. IPC-derived Dilps bind to the insulin receptor in the prothoracic gland to promote the production of ecdysone [32], demonstrating the tight coordination between growth rate and duration during larval growth. This crosstalk between insulin and ecdysone signaling to regulate both growth rate and duration is crucial in the size determination of the larva once it initiates pupariation.

Oxygen availability has been shown to be a key regulator of cell, tissue, and organismal growth. In mammalian cells, hypoxia inducible factors (HIFs) are transcriptional regulators of the molecular response to low oxygen concentrations [33]. The Drosophila HIF1-α homologue, Sima, is regulated post-translationally by protein stability and subcellular localization [33]–[37]. In normoxia, the HIF prolyl-hydroxylase, Fatiga, uses molecular oxygen to hydroxylate Sima, leading to its degradation [33], [34], [36]. However, hypoxic conditions prevent hydroxylation and degradation of Sima, increasing cytoplasmic levels with the subsequent increase in nuclear localization of the protein [33], [37]. Nuclear translocation of Sima during periods of low oxygen availability regulates the transcription and subsequent expression of a variety of genes that enable the organism to maintain homeostasis and survival [38], [39]. Examples of such target genes include those coding for specific glycolytic and lipolytic enzymes [39]–[41], as well as negative regulators of cell growth and proliferation [36], [42], [43]. Hypoxia has also been shown to modify numerous developmental processes, particularly tracheal sprouting and plasticity [44]–[46], illustrating tissue-level adaptations for survival under low oxygen concentrations.

Here, we show that rearing larvae under hypoxic conditions elicits lipid defects, including reduced fat body opacity and increased lipid droplet size, similar to those caused by starvation. However, unlike starvation, hypoxia does not cause lipids to accumulate in oenocytes, a group of hepatocyte-like cells that are thought to process lipids for energy during periods of stress [23], [47]. We present evidence that hypoxia reduces insulin receptor signaling by increased retention of Dilp2 in the larval IPCs, associated with larval growth inhibition. This growth restriction phenotype is rescued by increasing insulin receptor levels specifically in the larval tracheal system through augmentation of tracheal plasticity during hypoxia. Warts loss of function in the trachea can mimic insulin-mediated augmentation of tracheal plasticity under hypoxic conditions. Similar to the previously reported effects of hypoxia on ecdysone [5], we also observe an inhibition of proper epidermal and tracheal cuticle molting, both of which can be rescued by insulin signaling and Warts loss of function in the larval tracheal system. Finally, Warts loss of function in the trachea reverses the nuclear localization of Sima during hypoxia, reflecting increased oxygen delivery to peripheral tissues that reverses the growth restriction and molting defects induced by low oxygen availability. Our findings illustrate that while tracheal sprouting may increase during limited oxygen, hypoxia-associated reduction in insulin receptor signaling restricts tracheal growth and plasticity that are required for systemic larval growth during oxygen deprivation.

## Materials and Methods

### Drosophila stocks

The following stocks were used in this study: *w^1118^* (for most wildtype controls), *daughterless-GAL4 (da-GAL4), btl-GAL4 (breathless-GAL4, UAS-GFP), elav-GAL4, NP3084-GAL4, UAS-InR-WT, UAS-InR-RNAi, UAS-simaRNAi* (Bloomington Stock Center); *UAS-WartsRNAi* (Vienna Drosophila RNAi Center); *Dmef2-GAL4 (Dmef2-GAL4; UAS-mitoGFP)* (M. Guo); *R4-GAL4* (K. Bharucha); *phm22-GAL4,* (L. Fessler); *promE(800)-GAL4* (J. Levine); *UAS-Sima* (P. Wappner); *UAS-Gbb* (K. Broadie); UAS-ptkv* (E. Ferguson).

### Starvation and hypoxia experiments

Unless otherwise noted, all larvae in this study were staged according to the following procedure. Embryos were collected on grape juice plates. Larvae were collected within 3 hours after hatching and placed on standard Drosophila media until being transferred to starvation plates or into a hypoxia chamber. For starvation experiments [48], larvae were transferred to agar plates containing PBS/1% sucrose 48 hrs after hatching (hAH) and dissected 24 hrs thereafter. Fed controls were reared on standard *Drosophila* media. Hypoxia experiments were conducted using a hypoxia chamber (Coy Laboratory Products, MI). The appropriate hypoxic oxygen concentration was obtained as previously described [49], by mixing nitrogen with ambient air. For hypoxia experiments, unless otherwise noted, larvae were collected within 3 hAH, reared in normoxic conditions at 25°C from 0–24 hAH, transferred to a room temperature (RT) hypoxia chamber at 3.5% O_2_, and dissected after 48 hours. Dissections were performed immediately after removal from the chamber. In these experiments, normoxic controls were also reared at 25°C in ambient air from 0–72 hAH. Larval growth assessments were performed at 72 hAH by immobilizing on ice, obtaining light micrographs, and using ImageJ to calculate larval lengths and larval volumes (area of larva multiplied by its diameter). All statistical analyses were performed using a Student's t-test.

### Oil-Red-O staining

Oil-Red-O staining procedure was adapted from [23]. Oil-Red-O staining of the oenocytes was performed by partially dissecting larvae in 3.7% formaldehyde to expose the inner face of the epidermis and fixing for 10 minutes at RT. Oil-Red-O staining of the fat body and trachea was performed by dissecting larvae in 3.7% formaldehyde and fixing for 25 minutes. Tissues were rinsed twice with distilled water, incubated for 30 minutes in Oil-Red-O stain (3 ml of 0.1% Oil-Red-O [Alfa Aesar, MA] in isopropanol and 2 ml distilled water), and rinsed twice with distilled water. Tissues were mounted in Vectashield (Vector Labs, MI). Bright-field micrographs of Oil-Red-O-stained fat bodies and oenocytes were obtained using a Zeiss AX10 microscope.

### Immunohistochemistry

Tissues were dissected 72 hAH in 3.7% formaldehyde and fixed for 25 minutes at RT. Tissues were washed in 0.4% Triton X-100 in 1X PBS (0.4% PBT), 3X 15 minutes, and blocked in 10% NGS in 0.4% PBT at RT for 1–2 hours. Tissues were incubated in primary antibody in 0.4% PBT at 4°C overnight [1∶100 rat-anti Sima (P. Wappner), 1∶100 rabbit-anti phospho-Mad (E. De Robertis), 1∶100 mouse-anti Histone (Millipore)], then washed extensively in 0.4% PBT and incubated in secondary antibody in 10% NGS +0.4% PBT at 4°C overnight or 2 hours RT [mouse-FITC, rat-FITC, rabbit-Cy3 (Jackson Immunochemicals, PA)]. Tissues were again washed, incubated in 1∶500 TO-PRO3 (TOPRO; Invitrogen, CA) in distilled water for 30–60 minutes at RT (if noted), and mounted in Vectashield (Vector Laboratories, MI). Images were obtained using a Zeiss LSM5 confocal laser scanning microscope.

The procedure for Dilp2 analysis was adapted from [20], and is the same as outlined above but with the following exceptions: 5% BSA in 0.4% PBT was used instead of 10% NGS in 0.4% PBT. Primary antibody was 1∶100 rat-anti Dilp2 (P. Leopold) and secondary antibody was rat-Cy3 (Jackson Immunochemicals, PA).

### Tracheal phenotypic analyses

Visualization of tracheal branching was performed by placing larvae in a drop of Halocarbon oil 700 (Sigma) on a glass slide and heat immobilizing them on a hot plate for about 4 seconds. Bright-field micrographs were obtained immediately after using a Zeiss AX10 microscope. Visualization of defects in tracheal molting was performed by dissecting specimens in 3.7% formaldehyde along the ventral midline and fixing for 25 minutes at RT. Larval trachea were then mounted in Vectashield. Nomarski (DIC) and fluorescence micrographs were obtained using a Zeiss AX10 microscope. For quantitative analysis, we restricted our focus to the dorsal branch of the third tracheal segment, which is comprised of a main tracheal branch from which straight cellular processes extend (named “Thick Terminal Branches [TTBs]”) and thinner extensions project, as described by [45]. Number of TTBs were quantified for tracheal phenotypes.

## Results

### Hypoxia induces lipid metabolism adaptations that differ from those observed during starvation

**Figure 1:**
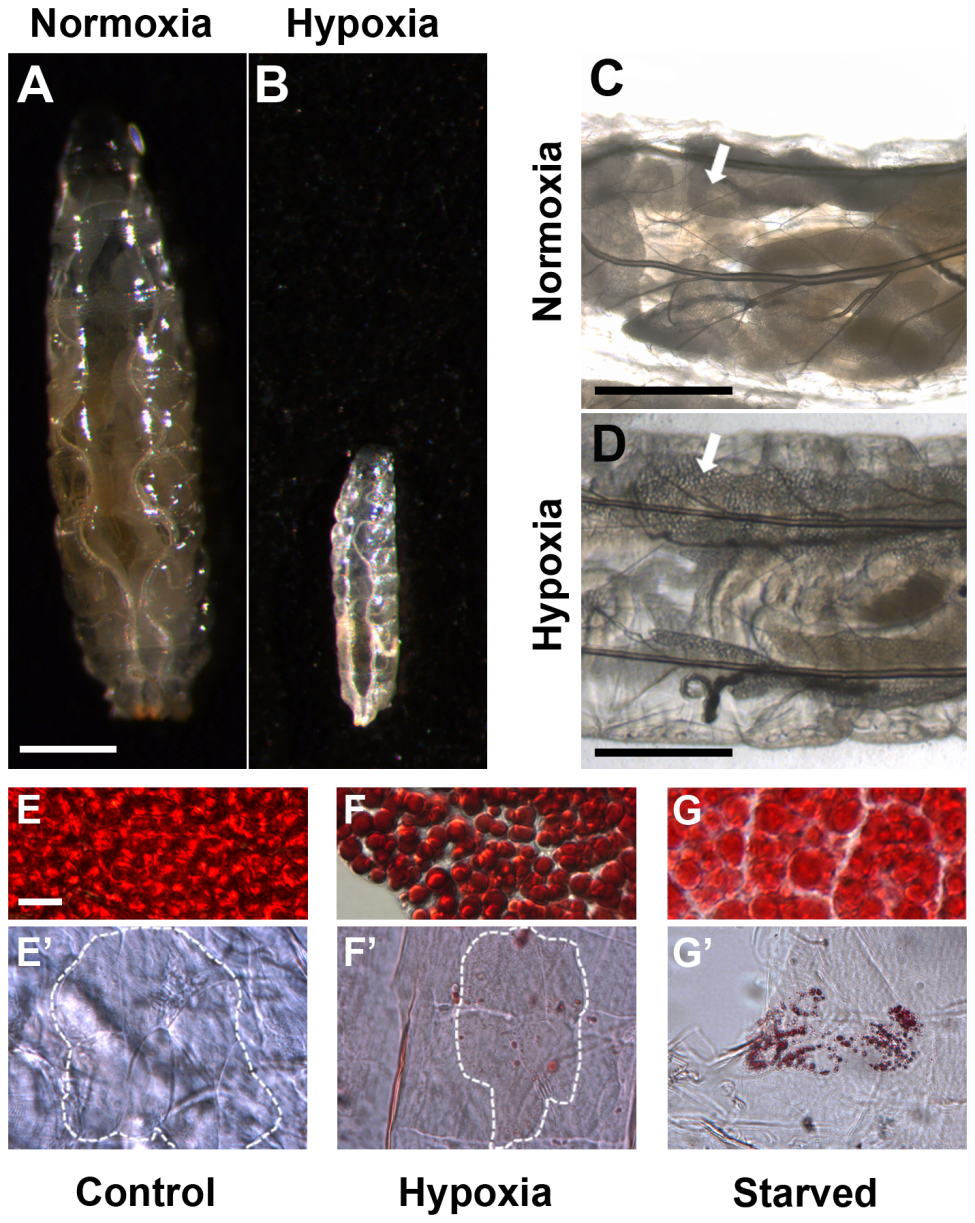
Hypoxia restricts larval growth and induces lipid metabolic changes distinct from starvation. [**A–B**] Rearing wildtype larvae under hypoxic conditions resulted in a significant reduction in larval size (**B**), compared to those reared under normoxic conditions (**A**). [**C–D**] Hypoxia decreased fat body opacity (**D**), as compared to that observed in normoxia (**C**). White arrows point to the larval fat body. [**E–G′**] Control larvae reared under hypoxic (*promE(800)>2xEGFP*) and starvation (*w^1118^*) conditions exhibited increased lipid droplet accumulation in the larval fat body (**F, G**), compared to the control (**E**). Lipids did not aggregate in the larval oenocytes during hypoxia (**F′**), compared to the control (**E′**), unlike what was observed during starvation (**G′**). Oenocytes in **E′** and **F′** are outlined with a dashed line, and their corresponding cellular structure can be appreciated in **S1A–B Fig**. Scale bar in **A** applies to **A–B**: 0.50 mm. Scale bar in C represents 0.50 mm, and scale bar in D represents 0.25 mm. Scale bar in E applies to E–G′: 20 µm.

Previous studies have demonstrated that hypoxic conditions imposed throughout the lifespan of the fly and manipulation of genes involved in the hypoxic response lead to an inhibition of growth at the cell, tissue, and organismal levels [2]–[5], [36], [43]. Rearing wildtype Drosophila larvae under hypoxic conditions leads to a reduction in body size (**Fig 1B** **; compare to 1A** and [5]). Given that starvation of flies also results in the reduction of size in cells, tissues, and entire organisms [18], and that both oxygen and nutrient availability are two key environmental cues that modulate growth, we compared the phenotypic consequences that arise under hypoxic conditions to those in starvation.

Prominent amongst the consequences of starvation are distinct changes in the fat body, a primary larval organ responsible for lipid metabolism. Proper lipid metabolism is crucial for the maintenance of organismal growth during periods of environmental stress, such as starvation, as the lipolysis of triglycerides stored in adipocyte fat droplets provides energy required for growth [23]. In Drosophila, lipids are stored inside intracellular droplets in larval fat body cells. Under normal feeding conditions, nutrients accumulate in larval fat body cells and cause the tissue to become opaque. However, during starvation, the opacity of the tissue decreases as lipids are mobilized from the fat body [48], [50] and accumulate in oenocytes [23]. Rearing wildtype larvae under hypoxic conditions resulted in decreased fat body opacity (**Fig. 1D** **; compare to 1C**), similar to that previously observed in a nutrient-deprived fat body [48]. Oil-Red-O staining of larval fat bodies showed that hypoxia caused lipids in this tissue to aggregate in larger droplets (**Fig. 1F**), as compared to those observed in the wildtype control (**Fig. 1E**). This same increase in lipid droplet aggregation was observed during starvation (**Fig. 1G** and [18], [23]). However, hypoxia caused the loss of distinct cell boundaries in the larval fat body while maintaining homogeneity in lipid droplet size, as compared to a starved fat body. Given that larger lipid vesicle aggregation in the fat body is an indication of efflux of lipids into the hemolymph [23], we investigated whether these lipids mobilized from the fat body were being taken up from circulation during oxygen deprivation by the larval oenocytes. Staining for lipids in the oenocytes of control larvae reared in hypoxia showed no accumulation of lipids in these cells (**Fig. 1F** **′**), as compared to the normoxic control (**Fig. 1E** **′**). The presence of intact oenocytes was confirmed by using an oenocyte-specific driver, *promE(800)-GAL4* [51], to drive the expression of a *2xEGFP* reporter. Expression of the reporter was observed in the oenocytes of larvae reared in either normoxia or hypoxia (**S1A–B Fig.**). This absence of lipid accumulation in the larval oenocytes differed from what is observed during starvation (**Fig. 1G** **′** and [23]). Moreover, hypoxia increased nuclear localization of Sima in the fat body (**S1D–D″ Fig.** **; compare to** **S1C–C″ Fig.**), and overexpression of Sima in this tissue, which induces a hypoxic response, resulted in increased lipid droplet accumulation in the fat body (**S1F Fig.** **; compare to** **S1E Fig.**) without eliciting lipid accumulation in the oenocytes (**S1H Fig** **; compare to** **S1G Fig.**).

This unique phenotypic combination of reduced fat body opacity, lipid aggregation in the fat body, and lack of lipid accumulation in the larval oenocytes exhibited by hypoxic larvae is reminiscent of those in gbb mutants [13]. This led us to examine whether a reduction in TGF-beta signaling was responsible for the defects in lipid metabolism by assessing the nuclear localization of the phosphorylated form of Mad (pMAD), a target of TGF-beta signaling [52]. Under hypoxic conditions, endogenous nuclear localization of pMAD was decreased (**S1J–J″ Fig.** **; compare to** **S1I–I″ Fig.**). Fat body-specific overexpression of an activated form of a Gbb receptor, thickveins, which drives nuclear localization of Mad (**S1K–L Fig.**), did not rescue growth restriction (**S1M Fig.**), and ubiquitous overexpression of Gbb failed to reverse the increased aggregation of lipid droplets in the fat body during hypoxia (**S1N–Q Fig.**). Taken together, these data demonstrate that hypoxia induces lipid metabolism defects associated with impaired BMP signaling, but in a manner distinct from that during starvation.

### Overexpression of the insulin receptor ubiquitously and in the larval trachea during hypoxia renders larvae resistant to hypoxic growth restriction

**Figure 2:**
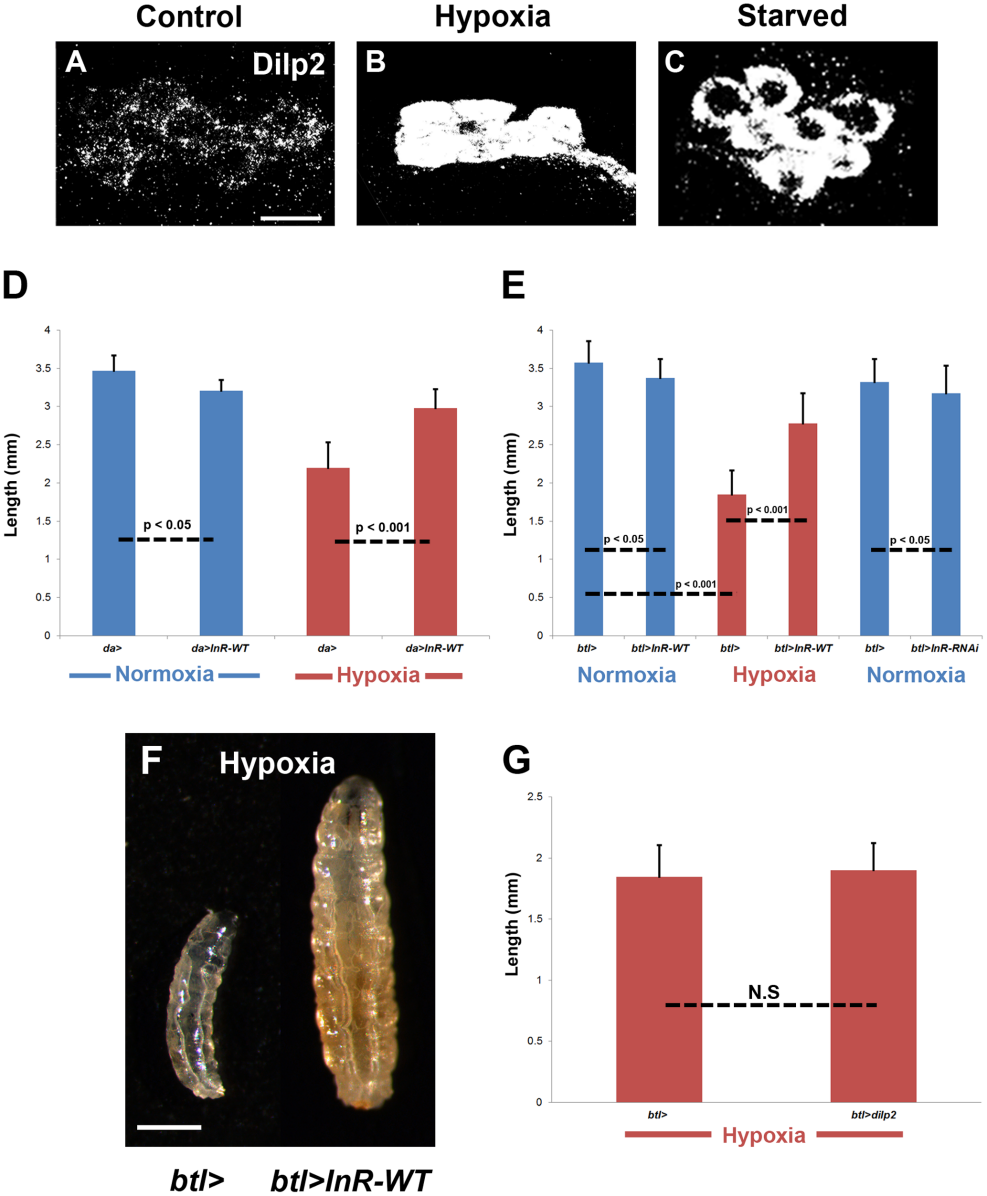
Insulin signaling under hypoxic conditions. [A–C] Dilp2 antibody staining (white) of the larval brain was hardly detected in the IPCs of wildtype larvae reared in normoxia (A) but accumulated in those of wildtype larvae reared in hypoxia (B). The increased Dilp2 retention under hypoxic conditions phenocopied that observed during starvation (C). IPCs in B were reared in 2.5% O_2_ from 48–72 hAH. [D] Under hypoxic conditions, ubiquitous overexpression of the wildtype form of the *Drosophila* insulin receptor (InR-WT), using the driver da-GAL4, led to a statistically significant rescue in larval size (n = 52), as compared to the hypoxic wildtype control (da>) (n = 49). Ubiquitous overexpression of the InR-WT led to a minor reduction in larval size under normoxic conditions (n = 37), as compared to the normoxic wildtype control (n = 33), most likely due to an increased growth rate and shortened duration of growth due to insulin-stimulated ecdysone production in the prothoracic gland [19], [24]–[26]. [E-F] InR-WT overexpression in the larval trachea, using the trachea-specific driver btl-GAL4, led to a statistically significant rescue of larval size during hypoxia (n = 20), as compared to the hypoxic wildtype control (btl>) (n = 30) (F; quantified in E). Under normoxic conditions, tracheal-specific overexpression (n = 20) of the insulin receptor leads to a statistically significant mild decrease in larval growth, as compared to the normoxic wildtype control (*btl>*) (n = 20). Tracheal-specific downregulation of the insulin receptor (n = 46) with its respective control (n = 54) was carried out at 29°C to enhance InR knockdown and leads to a statistically significant mild reduction in larval length. [G] Overexpression of Dilp2 in the larval trachea (btl>dilp2) under hypoxic conditions (n = 35) did not rescue growth restriction, as compared to the hypoxic wildtype control (btl>) (n = 20). Volumetric analysis also demonstrated no statistical significance (data not shown). Scale bar in A applies to A–C: 10 µm. Scale bar in F represents 0.50 mm. Statistical significance was determined using a Student's t-test.

We next examined whether oxygen availability regulates the secretion of specific Dilps from the IPCs of the larval brain, as starvation reduces circulating Dilp2 by inhibiting its secretion from the IPCs (**Fig. 2C** and [20]). Similar to what is observed during starvation, rearing wildtype larvae under hypoxic conditions increased the retention of Dilp2 in the larval IPCs (**Fig. 2B** **; compare to 2A**). Interestingly, overexpression of Sima in the larval fat body, which mimics the hypoxia-induced accumulation of Sima in this tissue (**S1C–D″ Fig.**), also resulted in increased retention of Dilp2 in the IPCs (**S2B Fig.** **; compare to** **S2A Fig.**). Conversely, downregulation of Sima in the larval fat body under oxygen deprivation alleviated the hypoxia-induced retention of Dilp2 in the IPCs (**S2F Fig.** **; compare to** **S2D Fig.**), in contrast to normoxic conditions in which downregulation of Sima does not affect Dilp2 secretion (**S2E Fig.** **; compare to** **S2C Fig.**). These data suggest that accumulation of Sima in the larval fat body during hypoxia non-autonomously prevents Dilp2 from being secreted from the brain.

Our findings suggest that hypoxia-dependent increased retention of Dilp2 peptides inhibits systemic growth by reducing insulin receptor signaling in peripheral tissues. Next, we asked if hypoxic growth restriction results from a ubiquitous reduction of insulin receptor signaling or from a reduction in a specific larval tissue. We initially attempted to rescue hypoxic growth restriction by ubiquitous overexpression of specific Dilps. Interestingly, ubiquitous overexpression of Dilp2 under normal conditions results in complete larval lethality (data not shown). Since overexpression of Dilp2 specifically in developing wing imaginal discs results in semi-lethality [53], we hypothesize that Dilp levels must be tightly regulated. Consistent with this hypothesis, ubiquitous overexpression of Dilps 1 and 3–6 increases larval lethality exclusively under hypoxic conditions but not under normoxic conditions (data not shown). Surprisingly, however, ubiquitous overexpression of the wildtype form of the Drosophila insulin receptor (InR-WT) under hypoxic conditions rescues growth restriction as assessed by larval length (**Fig. 2D**). Assessing growth through quantification of larval volume confirmed this rescue in larval size upon ubiquitous overexpression of the insulin receptor under hypoxic conditions (**S3A Fig.**). Although hypoxia increased the retention of Dilp2 in the larval IPCs (**Fig. 2B**), the ability of insulin receptor overexpression to rescue growth restriction suggests that oxygen deprivation does not result in a total retention of Dilp2, permitting the secretion of enough peptides into the hemolymph capable of promoting insulin receptor signaling in target cells.

We next investigated if a specific larval tissue could mediate the rescue of hypoxic growth restriction upon insulin receptor overexpression focusing on larval tissues previously shown to modulate organismal size. Specifically, overexpression of the insulin receptor in the fat body (R4-GAL4; [18]–[22]), prothoracic gland (phm22-GAL4; [19], [24]–[26]), muscle (Dmef-GAL4; [28]), gut (NP3084-GAL4; [29]), brain (elav-GAL4; [16], [17]), and oenocytes (promE(800)-GAL4; [23]) did not robustly increase larval size relative to the wildtype hypoxic control (**S4A–FH Fig.**). Interestingly, while overexpression of the insulin receptor specifically in the larval trachea using btl-GAL4 mildly affected larval size under normoxic conditions (**Fig. 2E**), it did rescue larval growth restriction observed in hypoxia, as assessed through both larval length (**Fig. 2E–F**) and volume (**Fig. S3B**). Additionally, although hypoxic growth restriction is rescued, aggregation of lipid droplets in the fat body is not alleviated upon tracheal-specific overexpression of the insulin receptor (**S5B Fig.** **; compare to** **S5A Fig.**). In contrast, trachea-specific downregulation of the insulin receptor alone under normal oxygen concentrations only mildly decreases overall body size (**Fig. 2E**), suggesting that reduced insulin signaling in the trachea in combination with low oxygen availability act synergistically to restrict organismal growth. We next examined if the reduction in insulin signaling results from decreased availability of Dilps that bind to the receptor specifically in the larval trachea. However, overexpression of Dilp2 in the larval trachea does not rescue hypoxic growth restriction (**Fig. 2G**), suggesting that hypoxia limits insulin receptor signaling. Taken together, these data demonstrate that hypoxic growth restriction stems from insufficient insulin receptor signaling in the larval trachea.

### Insulin signaling and loss of warts function enhance tracheal plasticity under hypoxia conditions

**Figure 3:**
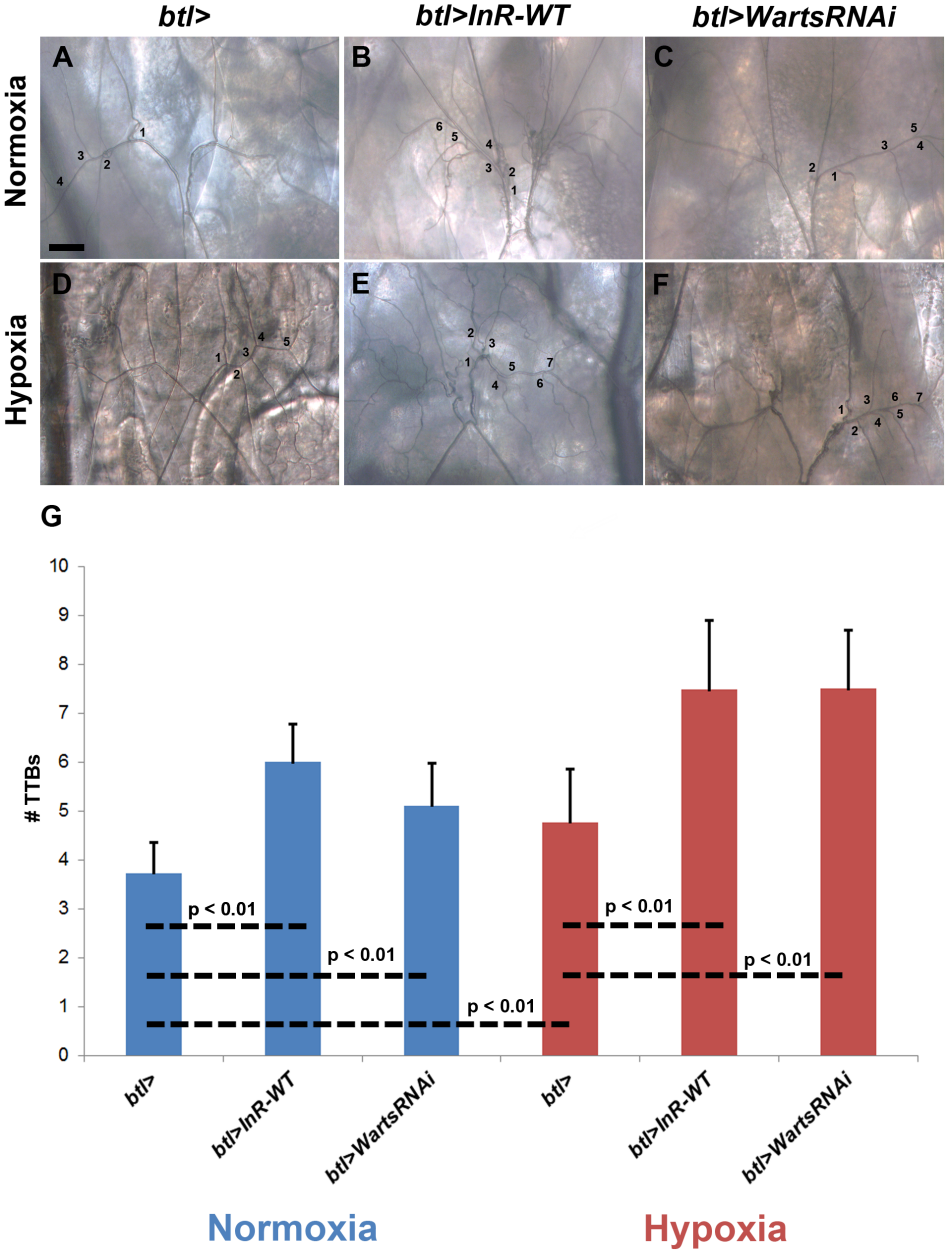
Insulin signaling and loss of Warts function augment the plasticity of the larval tracheal system. Overexpression of the wildtype insulin receptor (B) (n = 18) and downregulation of Warts (C) (n = 19) in the larval trachea increased tracheal growth and branching under normoxic conditions, compared to the normoxic wildtype control (A) (n = 22). Under hypoxic conditions, overexpression of the wildtype InR (**E**) (n = 23) and downregulation of Warts (**F**) (n = 18) in the larval trachea further increased the branching of this tissue, compared to the hypoxic control (**D**) (n = 21). Hypoxic conditions elicited an increase in tracheal sprouting (**D**), compared to the normoxic control (**A**). Scale bar in A applies to **A–F**: 50 µm. Numbers in **A–F** label terminal tracheal branches (TTBs), which are quantified in **G**.

Previous reports have demonstrated that tracheal cells sense and respond to oxygen levels and that hypoxia provokes increased sprouting of the Drosophila tracheal system [44], [45]. Rearing larvae under hypoxic conditions elicited a significant increase in tracheal sprouting (**Fig. 3D** **, compare to 3A; 3G**). Upon closer examination of hypoxic larval trachea, we observed that overexpression of the insulin receptor in the tracheal system resulted in an increased tracheal tortuosity phenotype after being subjected to oxygen deprivation (**Fig. 3E** **; compare to 3D**). A lesser degree of tortuosity upon overexpression of insulin receptor in the larval trachea was observed in normoxic conditions (**Fig. 3B** **; compare to 3A**). Interestingly, the increased tortuousness that resulted upon insulin receptor overexpression in the larval trachea is similar to that observed in Warts loss of function tracheal clones [54]. Warts encodes a serine/threonine kinase involved in the suppression of cell growth [55], [56]. Given the similarities in tracheal morphology, we hypothesized that downregulation of Warts in the larval trachea would result in enhanced tortuosity, as observed upon trachea-specific overexpression of the insulin receptor under hypoxic conditions. Knockdown of Warts in the larval trachea under hypoxic conditions increased tortuosity (**Fig. 3F** **; compare to 3D**), as compared to that in normoxic trachea (**Fig. 3C** **; compare to 3A**). Quantification of thick terminal branches (TTBs) [45] demonstrated that the number of TTBs increases upon overexpression of the insulin receptor and downregulation of Warts in both normoxic and hypoxic conditions (**Fig. 3G**).Collectively, these results suggest that although hypoxia elicits tracheal sprouting [44], [45], modulation of insulin and Warts signaling can augment the plasticity of the larval tracheal system to undergo further outgrowth during times of limiting oxygen availability.

### Hypoxia-induced inhibition of tracheal and cuticle molting is rescued by trachea-specific overexpression of the insulin receptor and downregulation of warts

**Figure 4:**
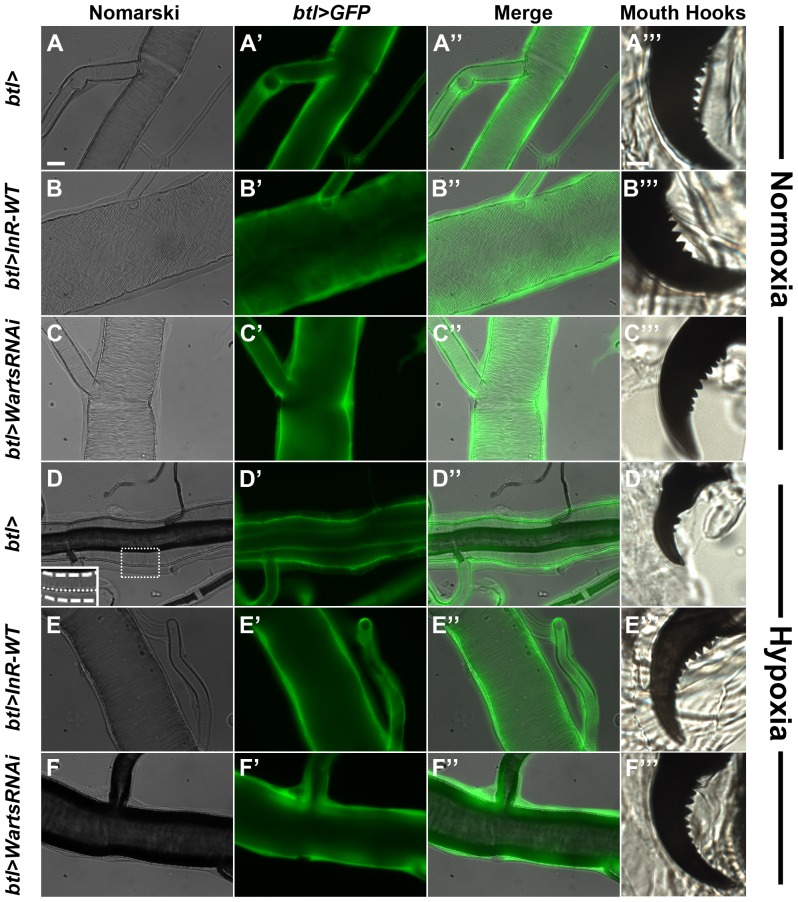
Hypoxia-induced molting defects are rescued by trachea-specific overexpression of the insulin receptor and Warts downregulation. Under normoxic conditions, overexpression of the wildtype InR (B–B″′) and downregulation of Warts (C–C″′) in the larval trachea did not inhibit molting of the larval trachea or progression through the larval instars, as demonstrated by the presence of third instar serrations in the mouth hooks. Normoxic wildtype controls are represented in A–A″′. Under hypoxic conditions, tracheal molting was inhibited (D–D″), and larvae did not reach the third instar developmental stage, as demonstrated by the presence of younger instar serrations in the mouth hooks (D″′). Inhibition of tracheal molting and developmental progression were rescued by tracheal-specific overexpression of the wildtype InR (E–E″′) and downregulation of Warts (F–F″′). Scale bar in A applies to A–F″′, excluding panels labeled as triple-primed (″′): 20 µm. Scale bar in A″′ applies to all triple-primed (″′) panels: 10 µm. Inset in D highlights persistence of early tracheal cuticles that had not been shed. The upper dashed line denotes the edge of the tracheal lumen that was inflated. The middle dotted line and bottom dashed line highlight the tracheal cuticles that had not been shed.

During the progression through the three larval instar stages, the steroid hormone ecdysone drives molting in which the larva sheds its cuticle in order to enable further increases in body size [6]. In conjunction with the shedding of the external cuticle at each larval molt, the tracheal system also sheds its own cuticle, which functions as structural support for maintaining the open airways of the larval trachea [57]. Failure to undergo molting will result in growth arrest [58]. Upon closer examination of hypoxic larval trachea, we observed defects in proper shedding of the tracheal cuticle (**Fig. 4D** **–D″; compare to 4A–A″**). Unlike starvation (data not shown), hypoxia also decreased the number of lipids that stud the larval trachea (**S5E Fig.** **; compare to** **S5D Fig.**). Additionally, larvae reared under hypoxic conditions exhibit defects in proper molting of the epidermal cuticle, as assessed by the presence of younger instar mouth hook serrations (**Fig. 4D″′;** **compare to 4A″′**). Interestingly, trachea-specific overexpression of the insulin receptor and downregulation of Warts during hypoxia rescue the defects in tracheal molting (**Fig. 4E–E″, 4F–F″;** **compare to 4B–B″, 4C–C″**) as well as proper progression through the larval epidermal molts (**Fig. 4E″′, 4F″′; compare to 4B″′, 4C″′**). Taken together, our findings demonstrate that hypoxia is associated with developmental delay in which hypoxic larvae cannot undergo proper epidermal and tracheal molting.

### Downregulation of warts in the larval trachea rescues hypoxic growth restriction and enhances oxygen delivery during oxygen deprivation

**Figure 5:**
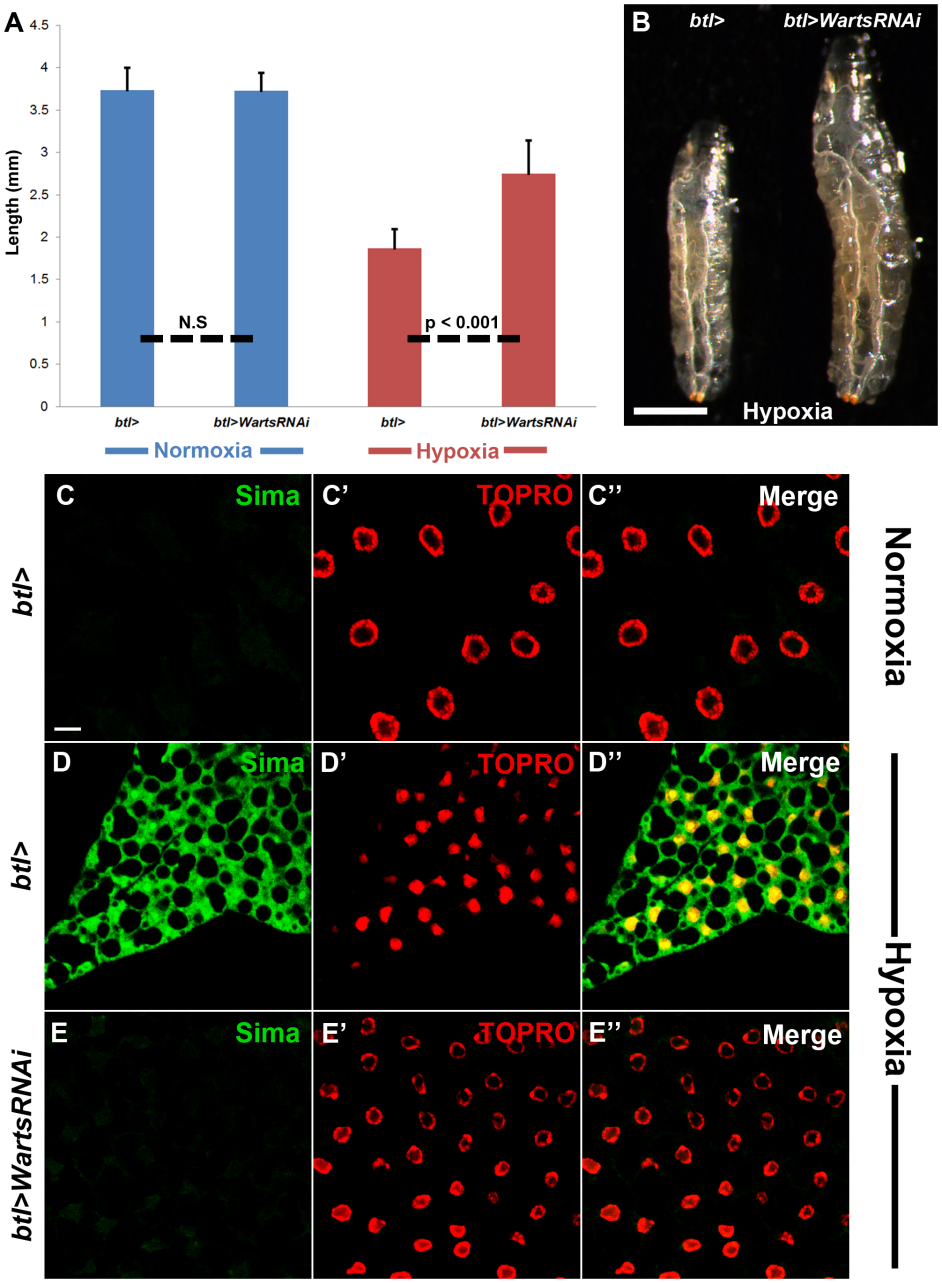
Warts downregulation in the larval trachea rescues growth restriction and enhances oxygen delivery. [A–B] Downregulation of Warts in the larval trachea led to a statistically significant increase in larval size under hypoxic conditions (n = 24), as compared to the hypoxic wildtype control (*btl>*) (n = 30). Downregulation of Warts in the trachea did not affect larval size under normoxic conditions (n = 30), as compared to the normoxic wildtype control (n = 17) (N.S denotes “no significance”). [C–E″] During normoxia, Sima (green) levels in the larval fat body were hardly detected (C–C″) compared to the significant increase in cytoplasmic and nuclear Sima under hypoxic conditions (D–D″). Upon downregulation of Warts in the larval trachea, Sima levels decrease in the fat body (E–E″), similar to those observed in the normoxic wildtype control (C–C″). TOPRO staining (red) marks cell nuclei. Scale bar in B represents 0.50 mm. Scale bar in C applies to C–E″: 20 µm.

Given the tracheal phenotypic similarities between overexpression of the insulin receptor and downregulation of Warts during hypoxia, we hypothesized that downregulation of Warts in the trachea could also rescue the growth restriction observed during oxygen deprivation. Indeed, similar to overexpression of the insulin receptor in this tissue, which renders larvae resistant to hypoxia-induced growth inhibition (**Fig. 2E–F**), downregulation of Warts in the tracheal system under hypoxic conditions increased larval length and volume, as compared to that in the hypoxic control (**Fig. 5A, 5B**; **S3C Fig.**). Additionally, similar to tracheal-specific overexpression of the insulin receptor under hypoxic conditions, downregulation of Warts in the trachea did not reverse hypoxia-induced lipid aggregation in the larval fat body (**S5C Fig.; compare to S5A Fig.**). We next evaluated whether downregulation of Warts in the trachea during hypoxia could lead to increased oxygenation of peripheral larval tissues, given its positive effects on tracheal tortuosity and larval growth. Rearing control larvae under hypoxic conditions resulted in increased nuclear localization as well as increased cytoplasmic detection of Sima in larval fat body cells (**Fig. 5D–D″**), as compared to control larvae reared in normal oxygen concentrations (**Fig. 5C**–5C″****). Interestingly, upon downregulation of Warts in the larval trachea, Sima levels returned to those observed in normoxic control larvae and nuclear localization was no longer detected (**Fig. 5E** **–5E″**). Given that Sima accumulation and localization to the nucleus are inhibited by the presence of molecular oxygen, these data demonstrate that modulation of Warts signaling and its effects on tracheal growth can enhance oxygen delivery to peripheral tissues during times when oxygen availability is limiting.

## Discussion

**Figure 6:**
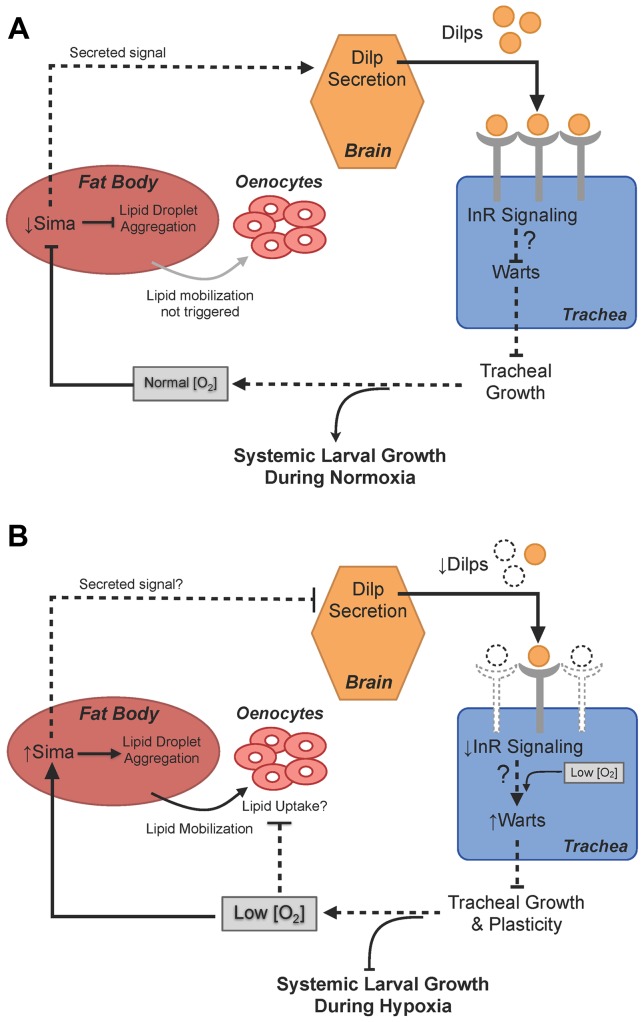
Lipid and insulin signaling changes in hypoxic larvae. [A] Under normoxic conditions, Sima is degraded and remains at low levels in the larval fat body. Increased lipid droplet aggregation does not occur, and lipids are not mobilized from the fat body into the hemolymph, eliminating the need for lipids to be taken up by the oenocytes. Under normal conditions, a fat-body derived signal is secreted and relayed to the larval brain in order to promote Dilp secretion from the insulin-producing cells [20], [22]. These secreted Dilps can go on to bind to the insulin receptor in target tissues to initiate insulin signaling. Activation of insulin signaling specifically in the larval trachea promotes tracheal growth, perhaps through an insulin-dependent inhibition of Warts. Under normoxic conditions, this feed-forward circuit involving fat body-mediated control of Dilp secretion and insulin- and Warts-dependent regulation of tracheal growth functions to modulate systemic growth during periods of adequate oxygen availability. [B] Under hypoxic conditions, degradation of Sima is reduced, and Sima accumulates in the larval fat body. Hypoxia-induced Sima accumulation in the fat body triggers increased lipid droplet aggregation in this tissue and lipid mobilization from the fat body into the hemolymph. Mobilization of lipids from the fat body is normally coupled with lipid uptake by the oenocytes. However, aggregation of lipid droplets is not observed under hypoxic conditions, suggesting a potential defect in lipid uptake by the oenocytes. Sima accumulation in the fat body results in increased Dilp2 retention, possibly through a reduction in the normal fat body-derived signal that promotes Dilp secretion from the IPCs. Increased Dilp2 retention in hypoxia is associated with the reduction of insulin signaling in peripheral tissues. As a consequence of systemic reduction of insulin receptor signaling to enable adaptation to hypoxia, the larval tracheal system is limited in its growth and plasticity, perhaps through increased Warts function.

Building upon previously reported findings associated with hypoxia-mediated reduction in organismal size [1]–[5], our results demonstrate distinct lipid metabolism defects elicited in hypoxia as well as a novel role for insulin signaling and Warts function in the adaptation to low oxygen availability. Under hypoxic conditions, Sima nuclear localization in the fat body is associated with lipid droplet aggregation and impaired lipid mobilization to oenocytes. The fat body in turn, perhaps through a secreted signal, inhibits Dilp2 secretion from the larval brain, associated with reduced insulin receptor signaling in peripheral tissues. Furthermore, decreasing Warts function mimics increased insulin receptor signaling in the trachea. These changes in signaling both rescue hypoxia-induced growth restriction and developmental delay through increased tracheal growth and plasticity, enhancing oxygen delivery during periods of oxygen deprivation (**Fig. 6**). Our findings demonstrate a mechanism that coordinates oxygen availability with systemic growth in which hypoxia-induced reduction of insulin receptor signaling decreases the plasticity of the larval trachea that is required for the maintenance of systemic growth during periods of oxygen deprivation.

### Metabolic adaptations triggered by hypoxia differ from those elicited by starvation

Hypoxic larvae display many of the same phenotypes as starved larvae, including systemic growth restriction, reduced fat body opacity, and lipid droplet accumulation in the fat body [18], [23], [48], [50]. However, while lipids aggregate in the larval oenocytes upon nutrient deprivation [23], this is not observed under hypoxic conditions. It has been proposed that the larval oenocytes are responsible for the processing of lipids that are mobilized from the fat body during periods of energy stress [23], [47]. The absence of lipid droplets in the oenocytes during hypoxia when lipids appear to be mobilized from the larval fat body suggests an impairment in lipid metabolism that is critical for energy generation and subsequent survival. Oenocytes may be oxygen-sensitive, only able to take up lipids mobilized from the fat body under adequate oxygen levels. Furthermore, inhibition of tracheal molting under hypoxic conditions may also be associated with the lack of lipid studding the larval trachea during oxygen deprivation, since deficits in fat body- and oenocyte-derived lipids affect proper cuticle formation in insects [59]. Very-long-chain fatty acids have also been implicated in waterproofing the larval trachea [60], [61], highlighting the role of proper lipid metabolism in maintaining tracheal integrity. The lack of lipid uptake in oenocytes, in contrast to starvation [23], during hypoxia may affect tracheal function and further limit oxygen delivery to peripheral tissues.

Furthermore, although hypoxic larvae display many of the hallmark phenotypes of Gbb mutants [13], in particular lipid droplet accumulation in the fat body without lipid aggregation in the oenocytes, ubiquitous overexpression of Gbb cannot rescue the hypoxia-induced lipid defects. It is possible that hypoxia inhibits signaling downstream of the TGF-beta/BMP receptors in larval tissues, in addition to the fat body, to elicit such lipid metabolism defects. This in turn may affect insulin signaling that perhaps is regulated by the TGF-beta pathway. Additional studies will establish the potential connections between TGF-beta/BMP and insulin signaling that regulate lipid homeostasis under hypoxic conditions.

### The larval trachea serves as a body size sensor under hypoxic conditions

Hypoxia stimulates tracheal terminal branches to bud towards oxygen-deprived tissues [44], [45]. This process is mediated by hypoxia-induced expression of Sima in the larval trachea, which promotes tracheal branching [44], [45]. Here, we show that additional growth of the larval trachea through activation of the insulin signaling pathway and downregulation of Warts under hypoxic conditions is sufficient to rescue growth restriction that stems from oxygen deprivation. Although hypoxia already elicits sprouting of the larval trachea [44], [45], increasing insulin signaling or loss of Warts function enhances plasticity of this tissue to combat the reduced delivery of oxygen to deprived tissues, thus promoting systemic larval growth. Furthermore, insulin signaling affects Sima nuclear localization [37] while Warts function influences epithelial tube size [54], demonstrating that these three pathways may converge to regulate tracheal growth and branching under hypoxic conditions.

Interestingly, body size in Manduca sexta larvae is regulated by a fixed tracheal system that can only deliver adequate oxygen supply to developing tissues for a specific duration of time during each instar stage [62]. As the organism grows in each instar stage, the fixed tracheal system of Manduca sexta larvae cannot meet the increasing demand for oxygen that results from the increase in body mass [62]. Thus, body size can only increase after each interinstar molt deposits a larger tracheal system that is able to deliver an adequate oxygen supply to a larger organism [62]. Similarly, under hypoxic conditions, Drosophila larval body size is constrained by the amount of tracheal growth, which may be limited by the reduction of insulin receptor signaling in the trachea.

Insulin signaling can promote tissue growth by reducing Warts activation [63]. Therefore, the reduction of insulin signaling under hypoxic conditions could lead to increased activation of Warts and limit tracheal plasticity. This is supported by our finding that downregulation of Warts is similar to trachea-specific activation of insulin signaling in both normoxia and hypoxia. Previous genetic epistatic experiments have demonstrated that cell growth and additional lumen formation caused by Warts loss of function in the tracheal system is dependent on blistered/pruned/SRF [54]. We surmise that tracheal plasticity is modulated during periods of oxygen deprivation through a decrease in Warts-dependent signaling, but is tempered by the hypoxia-induced limitation of insulin receptor signaling. Collectively, our findings demonstrate that proper modulation of insulin- and Warts-mediated signaling in the larval trachea is essential for the adaptation to low oxygen availability in order to accommodate organismal growth.

While our results indicate that insulin signaling and loss of Warts function in the larval tracheal system are sufficient to reverse hypoxic growth restriction by enhancing oxygen delivery, we cannot rule out the possibility that the trachea systemically regulates growth independent from its oxygen delivery functions. For instance, recent studies have shown that the trachea can release factors to regulate specific biological processes [64], [65]. Upon bacterial infection, the larval trachea produces and releases antimicrobial peptides (AMPs) into circulation as part of the organism's innate immune response [64]. Additionally, it has been demonstrated that the trachea produces a Decapentaplegic (Dpp) concentration gradient that activates Dpp signaling in enterocytes to control adult midgut homeostasis [65]. These findings suggest the possibility that hypoxia may inhibit tracheal growth factor production and/or release, which itself may be regulated by insulin and Warts inputs, in order to systemically restrict growth.

### Fine tuning of insulin signaling through receptor and ligand levels is required for the adaptive response to hypoxia

Systemic reduction of insulin signaling has been recently demonstrated to be an important adaptive response to stress. For example, under instances of muscle mitochondrial injury, muscle cells secrete ImpL2 [66] in order to systemically antagonize insulin signaling by binding to and inhibiting Drosophila insulin-like peptides [67]–[69]. Additionally, during starvation conditions where increased Dilp2 retention in the larval IPCs has been observed, forced membrane depolarization and neurosecretion of Dilp2 results in increased larval lethality under periods of nutrient deprivation [20], suggesting that excessive insulin signaling mediated by increased concentration of Dilps is toxic for the animal under stress conditions. In agreement with our findings, the increased Dilp2 retention in the larval IPCs that we observe under hypoxic conditions is most likely an adaptive mechanism to suppress insulin toxicity during periods of low oxygen availability. This increased retention of Dilp2 is complemented by the putative reduction of insulin receptor signaling in peripheral tissues such as the larval trachea, since overexpression of Dilp2 in the tracheal system under hypoxic conditions did not elicit the same organismal growth rescue as tracheal-specific overexpression of the insulin receptor. The combination of reduced circulating Dilps and decreased insulin receptor function in peripheral tissues demonstrates the coordination of a synergistic stress response mechanism to regulate insulin signaling under hypoxic conditions.

The increased toxicity of Dilps during limited oxygen availability is highlighted by the fact that ubiquitous overexpression of a majority of the Dilps (1, 3–6) under hypoxic conditions resulted in decreased survival. The detrimental effect of high Dilp2 levels is demonstrated by its lethal effects upon ubiquitous overexpression. In contrast, mild increases in insulin signaling mediated by overexpression of the insulin receptor promote the organism's adaptive response to hypoxia, as demonstrated by the hypoxic growth rescue that result upon overexpression of the insulin receptor ubiquitously and in the larval trachea. The simplest explanation is that increasing the number of insulin receptors available for binding enables the transduction of signaling with fewer circulating ligands, while excessive amounts of ligands can overwhelm the insulin signaling cascade and act detrimentally during conditions of stress. Therefore, careful titration of insulin levels is essential for an organism's adaption to dynamic environmental inputs.

The root of insulin toxicity under hypoxic conditions may arise from its role in regulating metabolism. During hypoxia, cells shift towards glycolytic metabolism to support energy homeostasis [70]. Activation of insulin signaling, however, shifts cells away from hypoxia-induced anaerobic glycolysis and towards oxidative metabolism [71], [72], a transition that may become debilitating for an organism trying to adapt to hypoxia. Additionally, the degree of hypoxia can either trigger HIF-dependent autophagy as a molecular adaptation to energy stress by creating additional substrates for energy production [73] or elicit excessive autophagy that causes cell death [74], [75]. We hypothesize that extreme activation of insulin signaling mediated by the presence of high levels of circulating insulin peptides may interfere with the organism's metabolic program that is activated to adapt to hypoxia. Indeed, overexpression of Dilp2 in developing wing imaginal discs triggers excessive autophagy associated with larval lethality [53]. Thus, excessive insulin inputs drive cells towards a metabolic state that is not conducive for adaptation to hypoxic conditions, possibly leading to the insulin toxicity we observe in hypoxic larvae and highlighting the importance of the careful titration of insulin peptides during oxygen deprivation.

### Altered insulin signaling as a conserved adaptive response to hypoxia in humans

Our studies have established a conserved model of reduced insulin signaling under hypoxia conditions in Drosophila. Alterations in insulin signaling in humans are associated with a vast number of risk factors or co-morbid conditions including obesity, cardiovascular disease, and intermittent hypoxia conditions, such as obstructive sleep apnea. Intermittent or tissue hypoxia has been proposed to contribute to the etiology of impaired insulin signaling. Previous studies have proposed that reduced insulin signaling in obese humans could be the result of microhypoxia in overgrown adipose tissue [76]. Adipose tissue hypoxia has recently been proposed as a mechanism for limited insulin signaling in both obesity and obstructive sleep apnea [77]. Furthermore, conditioned medium from hypoxic adipocytes alters insulin signaling in muscle [78], suggestive of an inflammatory signal that may regulate systemic insulin inputs. In Drosophila, the closest homologues to cytokines are the family of Unpaired ligands, which signal through JAK-STAT receptors. HIF-1α has been shown to regulate STAT signaling to modulate insulin sensitivity [79]. This, coupled with the finding that Upd2 regulates Dilp secretion in Drosophila [22], further supports the conservation of coordinated insulin signaling in flies and humans as an adaptive response to environmental stressors, such as hypoxia.

## Supporting Information

S1 Fig Effects of Sima and TGF-beta signaling in hypoxia. [**A–B**] Expression of a *2xEGFP* reporter using an oenocyte-specific driver, *promE(800)-GAL4*, demonstrates the presence of oenocytes in larvae reared under hypoxic conditions (**B**), compared to those reared under normoxic conditions (**A**). [**C–D″**] Nuclear localization of Sima (green) increased in wildtype fat body cells when larvae were reared under hypoxic conditions (**D–D″**), compared to that observed in the normoxic wildtype control (**C–C″**). TOPRO (blue) stains cell nuclei. [**E–H**] Overexpression of Sima in the larval fat body, using the fat body driver *R4-GAL4*, increased lipid droplet accumulation in this tissue (*R4>sima*; **F**), compared to the wildtype control (*R4>*; **E**). Lipids did not aggregate in the larval oenocytes upon overexpression of Sima in the fat body (**H**), compared to the wildtype control (**G**). [**I–J″**] Rearing wildtype larvae under hypoxic conditions decreased nuclear localization of phospho-Mad (pMAD; red) in larval fat body cells (**J–J″**), as compared to the normoxic control (**I–I″**). Histone (His; green) marks cell nuclei. [**K–L**] Overexpression of an activated form of the thickveins receptor in the larval fat body (R4>ptkv*) increased nuclear localization of phospho-Mad (pMAD) under normoxic conditions (**L**), compared to the wildtype control (**K**). [**M**] Overexpression of the activated thickveins receptor in the larval fat body (R4>ptkv*) under hypoxic conditions did not increase larval size, compared to the hypoxic wildtype control (*R4>*). Statistical significance was determined through a Student's t-test. [**N–Q**] Ubiquitous overexpression of Gbb (*da>Gbb*) under hypoxic conditions (**Q**) did not reverse the increased lipid droplet accumulation in the fat body that is observed in the hypoxic control (*da>*; **P**). Oil-Red-O staining of fat body derived from larvae with ubiquitous overexpression of Gbb under normoxic conditions (**O**) was indistinguishable from the normoxic wildtype control (**N**). Larvae were placed in 29°C from 19–24 hAH prior to hypoxic (or normoxic) exposure. Scale bar in **A** applies to **A–B**: 20 µm. Scale bar in **C** applies to **C–D″**: 20 µm. Scale bar in **E** applies to **E–F**: 20 µm. Scale bar in **G** applies to **G–H**: 50 µm. Scale bar in **I** applies to **I–J″**: 20 µm. Scale bar in **K** applies to **K–L**: 50 µm. Scale bar in **N** applies to **N–Q**: 20 µm.
(TIF)

S2 Fig Sima in the fat body regulates Dilp2 secretion from the IPCs. [**A–B**] Overexpression of Sima in the larval fat body (*R4>sima*) increased Dilp2 (white) retention in the IPCs (**B**), compared to the wildtype control (*R4>*; **A**). Scale bar in **A** applies to **A–B**: 10 µm. [**C–F**] Sima knockdown in the larval fat body under hypoxic conditions reversed the hypoxia-induced Dilp2 retention in the brain (**F**), as compared to the hypoxic control (**D**), with a spectrum of phenotypes ranging from complete Dilp2 secretion to a partial Dilp2 release. Sima knockdown in the larval fat body under normoxic conditions did not affect Dilp2 secretion from the IPCs (**E**), compared to the normoxic control (**C**). Scale bar in **C** applies to **C–F**: 10 µm.
(TIF)

S3 Fig Volumetric analysis confirms changes in larval growth as assessed by larval length. [**A**] Ubiquitous overexpression of the wildtype form of the insulin receptor under normoxic conditions led to a minor though statistically significant decrease in larval volume (n = 37), as compared to the normoxic control (n = 33). Rearing control larvae (*da>*) under hypoxic conditions significantly reduced larval volume (n = 49), as compared to those reared under normoxic conditions. Ubiquitous overexpression of the wildtype insulin receptor under hypoxic conditions resulted in a statistically significant increase in larval volume (n = 52), as compared to the hypoxic control. Statistical significance was determined through a Student's t-test. [**B**] Tracheal-specific overexpression of the wildtype form of the insulin receptor under normoxic conditions led to a minor though statistically significant decrease in larval volume (n = 20), as compared to the normoxic control (n = 20). Rearing control larvae (*btl>*) under hypoxic conditions significantly reduced larval volume (n = 30), as compared to those reared under normoxic conditions. Tracheal-specific overexpression of the wildtype insulin receptor under hypoxic conditions resulted in a statistically significant increase in larval volume (n = 20), as compared to the hypoxic control. Downregulation of the insulin receptor in the trachea under normoxic conditions and at 29°C to enhance InR knockdown did not lead to a statistically significant change in larval volume (n = 20), as compared to its respective control (n = 20). Statistical significance was determined through a Student's t-test. [**C**] Tracheal-specific downregulation of Warts under normoxic conditions did not lead to a statistically significant change in larval volume, as compared to the normoxic control. Rearing control larvae (*btl>*) under hypoxic conditions significantly reduced larval volume, as compared to those reared under normoxic conditions. Downregulation of Warts in the trachea under hypoxic conditions resulted in a statistically significant increase in larval volume, as compared to the hypoxic control. Statistical significance was determined through a Student's t-test.
(TIF)

S4 Fig Survey of tissue-specific overexpression of the insulin receptor during hypoxia. [**A–F**] Under hypoxic conditions, overexpression of the wildtype insulin receptor in the fat body (*R4>*) (**A**), prothoracic gland (*phm22>*) (**B**), muscle (*dmef>*) (**C**), gut (*NP3084>*) (**D**), brain (*elav>*) (**E**), and oenocytes (*promE(800)*>) (**F**) did not lead to robust increases in larval size. Larvae in **A** were reared in 2.5% O_2_ from 42–72 hAH. Calculation of larval volumes for each genotype also did not show statistically significant differences (data not shown).
(TIF)

S5 Fig Lipid phenotypes in the fat body and trachea during hypoxia. [**A–C**] Overexpression of the insulin receptor (**B**) and downregulation of Warts (**C**) in the trachea did not reverse the hypoxia-induced lipid aggregation in the larval fat body, as determined by Oil-Red-O staining (**A**). Scale bar in **A** applies to **A–C**: 20 µm. [**D–E**] Under normoxic conditions, wildtype larvae exhibited lipid studding of their tracheal system (**D**), as visualized by Oil-Red-O staining. Under hypoxic conditions, lipid studding of the trachea was eliminated (**E**). Scale bar in **D** applies to **D–E**: 20 µm.
(TIF)
