# Supplementary figures and images for: Insulin- and Warts-Dependent Regulation of Tracheal Plasticity Modulates Systemic Larval Growth during Hypoxia in Drosophila melanogaster
Source: PLoS One. 2014 Dec 26;9(12):e115297. doi: 10.1371/journal.pone.0115297 (PMC4277339; doi:10.1371/journal.pone.0115297)

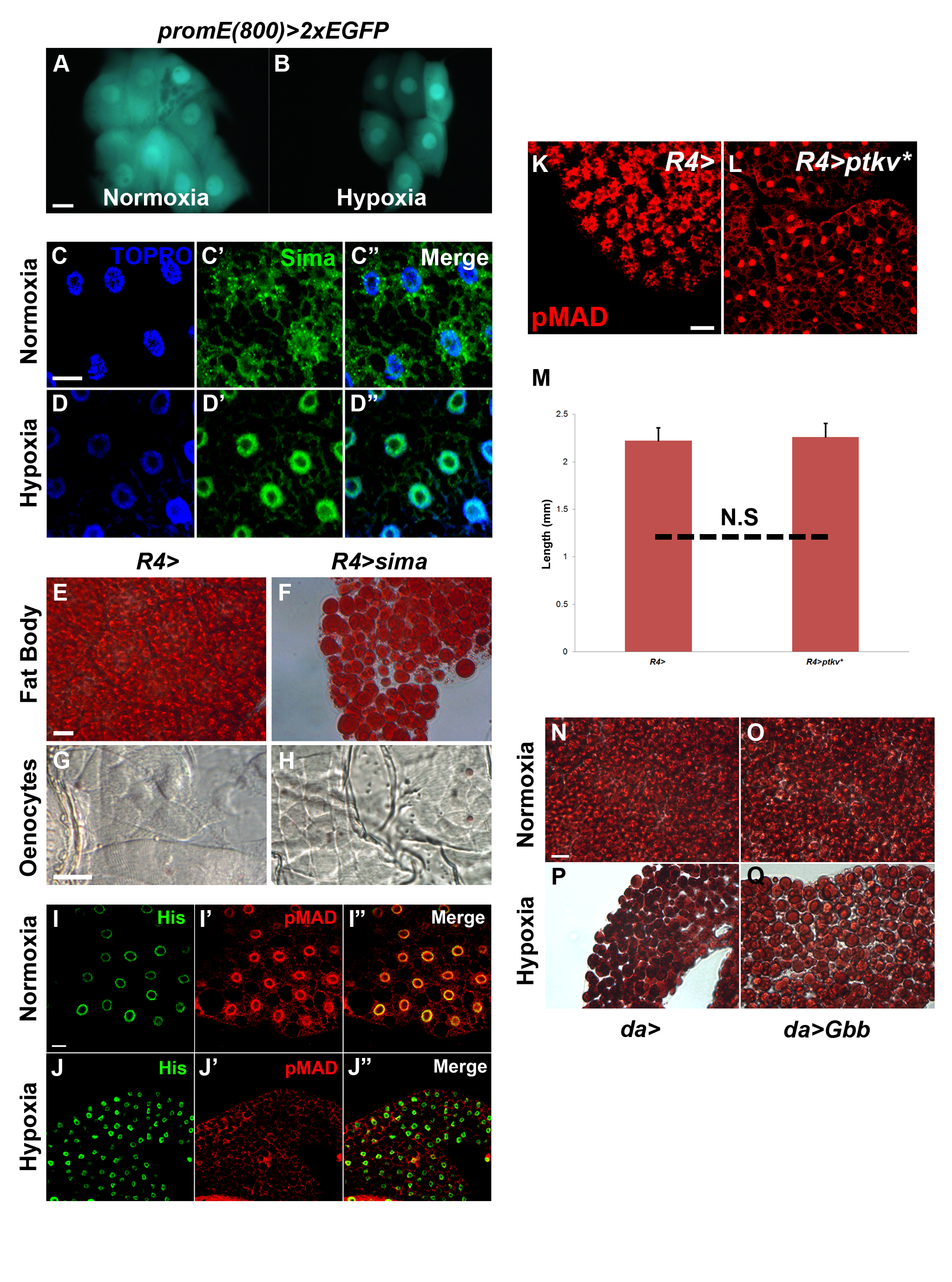

Supplement: S1 Fig — Effects of Sima and TGF-beta signaling in hypoxia. [A–B] Expression of a 2xEGFP reporter using an oenocyte-specific driver, promE(800)-GAL4, demonstrates the presence of oenocytes in larvae reared under hypoxic conditions (B), compared to those reared under normoxic conditions (A). [C–D″] Nuclear localization of Sima (green) increased in wildtype fat body cells when larvae were reared under hypoxic conditions (D–D″), compared to that observed in the normoxic wildtype control (C–C″). TOPRO (blue) stains cell nuclei. [E–H] Overexpression of Sima in the larval fat body, using the fat body driver R4-GAL4, increased lipid droplet accumulation in this tissue (R4>sima; F), compared to the wildtype control (R4>; E). Lipids did not aggregate in the larval oenocytes upon overexpression of Sima in the fat body (H), compared to the wildtype control (G). [I–J″] Rearing wildtype larvae under hypoxic conditions decreased nuclear localization of phospho-Mad (pMAD; red) in larval fat body cells (J–J″), as compared to the normoxic control (I–I″). Histone (His; green) marks cell nuclei. [K–L] Overexpression of an activated form of the thickveins receptor in the larval fat body (R4>ptkv*) increased nuclear localization of phospho-Mad (pMAD) under normoxic conditions (L), compared to the wildtype control (K). [M] Overexpression of the activated thickveins receptor in the larval fat body (R4>ptkv*) under hypoxic conditions did not increase larval size, compared to the hypoxic wildtype control (R4>). Statistical significance was determined through a Student's t-test. [N–Q] Ubiquitous overexpression of Gbb (da>Gbb) under hypoxic conditions (Q) did not reverse the increased lipid droplet accumulation in the fat body that is observed in the hypoxic control (da>; P). Oil-Red-O staining of fat body derived from larvae with ubiquitous overexpression of Gbb under normoxic conditions (O) was indistinguishable from the normoxic wildtype control (N). Larvae were placed in 29°C from 19–24 hAH prior [file pone.0115297.s001.tif]

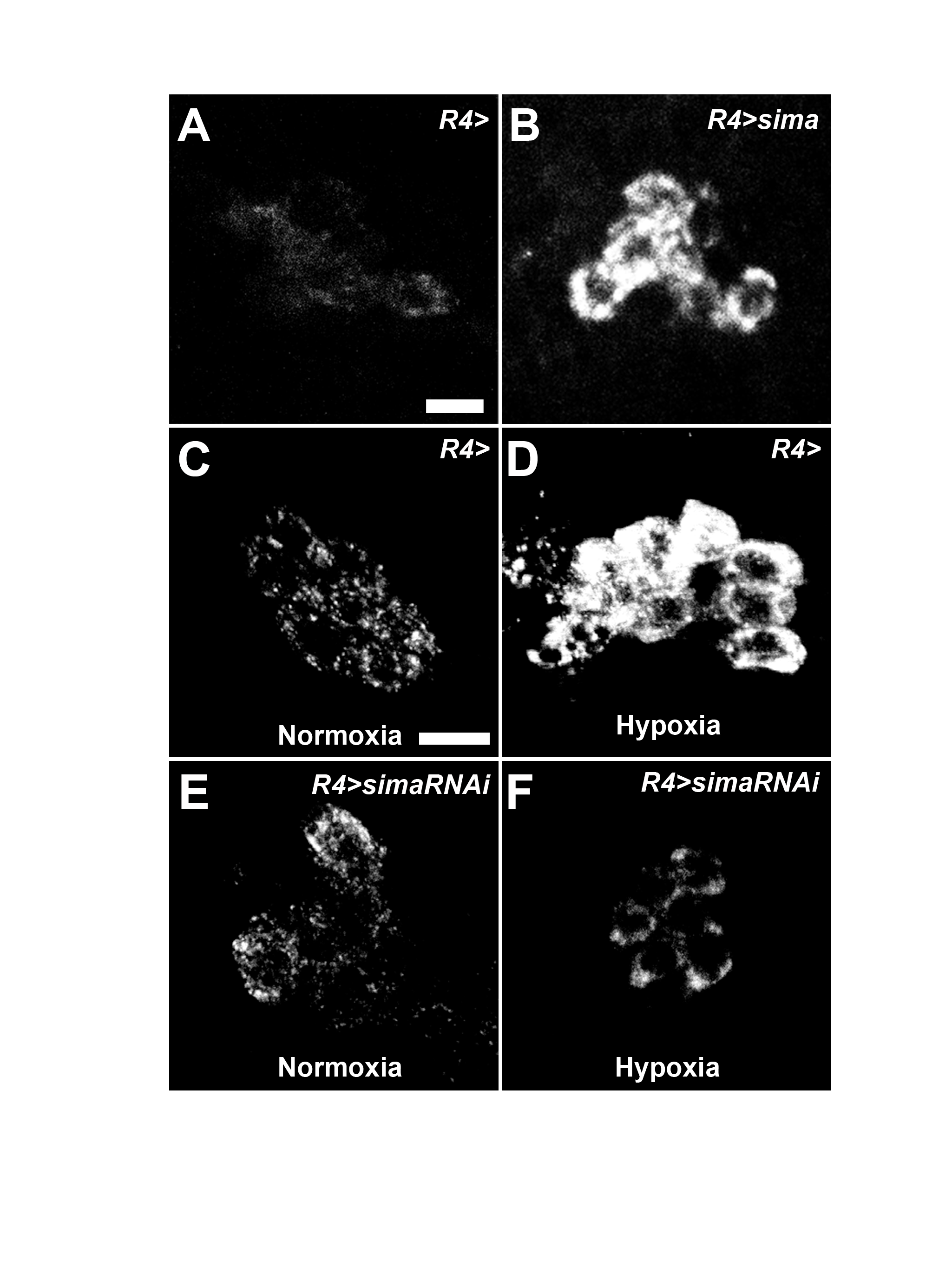

Supplement: S2 Fig — Sima in the fat body regulates Dilp2 secretion from the IPCs. [A–B] Overexpression of Sima in the larval fat body (R4>sima) increased Dilp2 (white) retention in the IPCs (B), compared to the wildtype control (R4>; A). Scale bar in A applies to A–B: 10 µm. [C–F] Sima knockdown in the larval fat body under hypoxic conditions reversed the hypoxia-induced Dilp2 retention in the brain (F), as compared to the hypoxic control (D), with a spectrum of phenotypes ranging from complete Dilp2 secretion to a partial Dilp2 release. Sima knockdown in the larval fat body under normoxic conditions did not affect Dilp2 secretion from the IPCs (E), compared to the normoxic control (C). Scale bar in C applies to C–F: 10 µm. (TIF) [file pone.0115297.s002.tif]

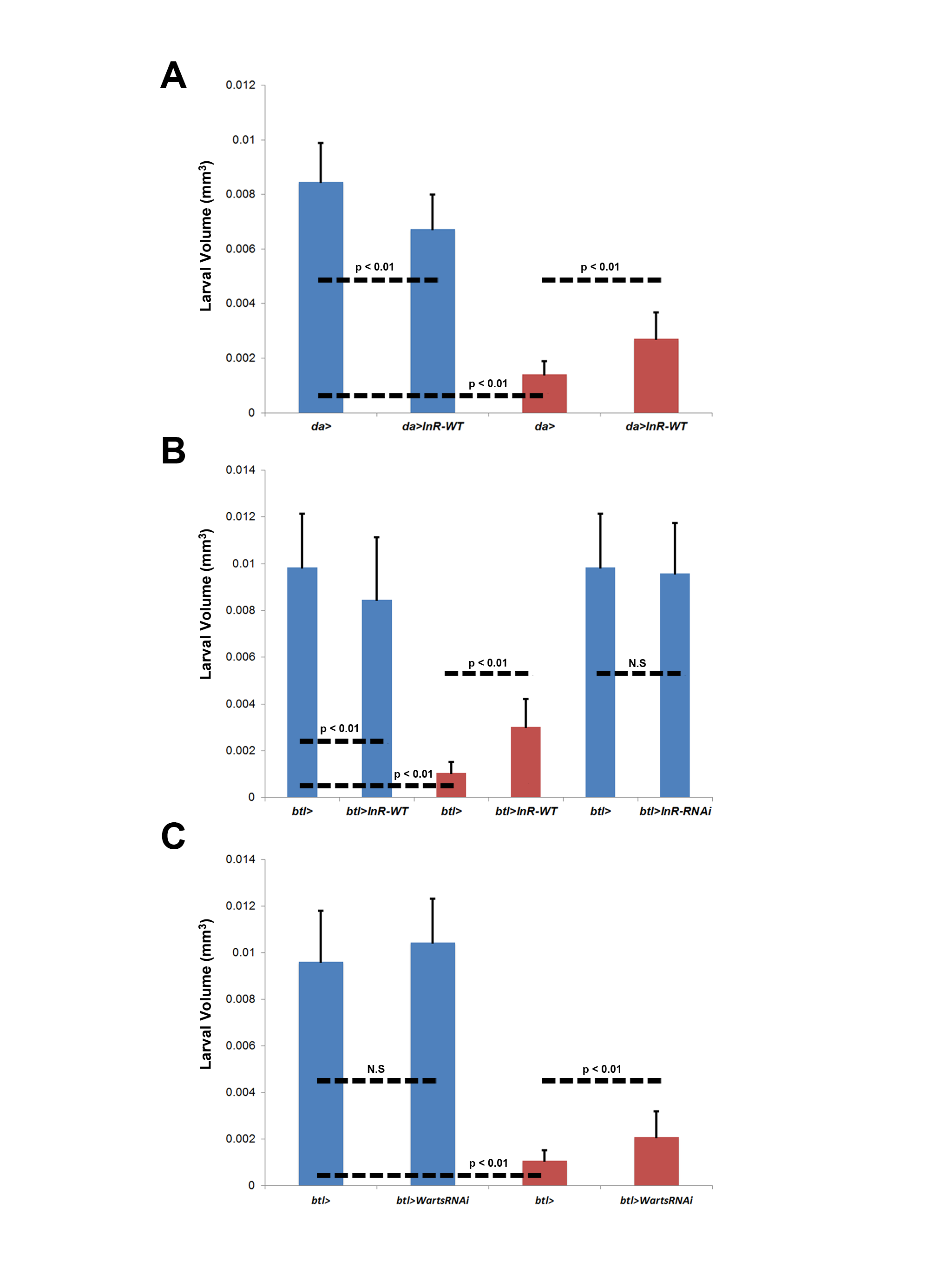

Supplement: S3 Fig — Volumetric analysis confirms changes in larval growth as assessed by larval length. [A] Ubiquitous overexpression of the wildtype form of the insulin receptor under normoxic conditions led to a minor though statistically significant decrease in larval volume (n = 37), as compared to the normoxic control (n = 33). Rearing control larvae (da>) under hypoxic conditions significantly reduced larval volume (n = 49), as compared to those reared under normoxic conditions. Ubiquitous overexpression of the wildtype insulin receptor under hypoxic conditions resulted in a statistically significant increase in larval volume (n = 52), as compared to the hypoxic control. Statistical significance was determined through a Student's t-test. [B] Tracheal-specific overexpression of the wildtype form of the insulin receptor under normoxic conditions led to a minor though statistically significant decrease in larval volume (n = 20), as compared to the normoxic control (n = 20). Rearing control larvae (btl>) under hypoxic conditions significantly reduced larval volume (n = 30), as compared to those reared under normoxic conditions. Tracheal-specific overexpression of the wildtype insulin receptor under hypoxic conditions resulted in a statistically significant increase in larval volume (n = 20), as compared to the hypoxic control. Downregulation of the insulin receptor in the trachea under normoxic conditions and at 29°C to enhance InR knockdown did not lead to a statistically significant change in larval volume (n = 20), as compared to its respective control (n = 20). Statistical significance was determined through a Student's t-test. [C] Tracheal-specific downregulation of Warts under normoxic conditions did not lead to a statistically significant change in larval volume, as compared to the normoxic control. Rearing control larvae (btl>) under hypoxic conditions significantly reduced larval volume, as compared to those reared under normoxic conditions. Downregulation of Warts in the tr [file pone.0115297.s003.tif]

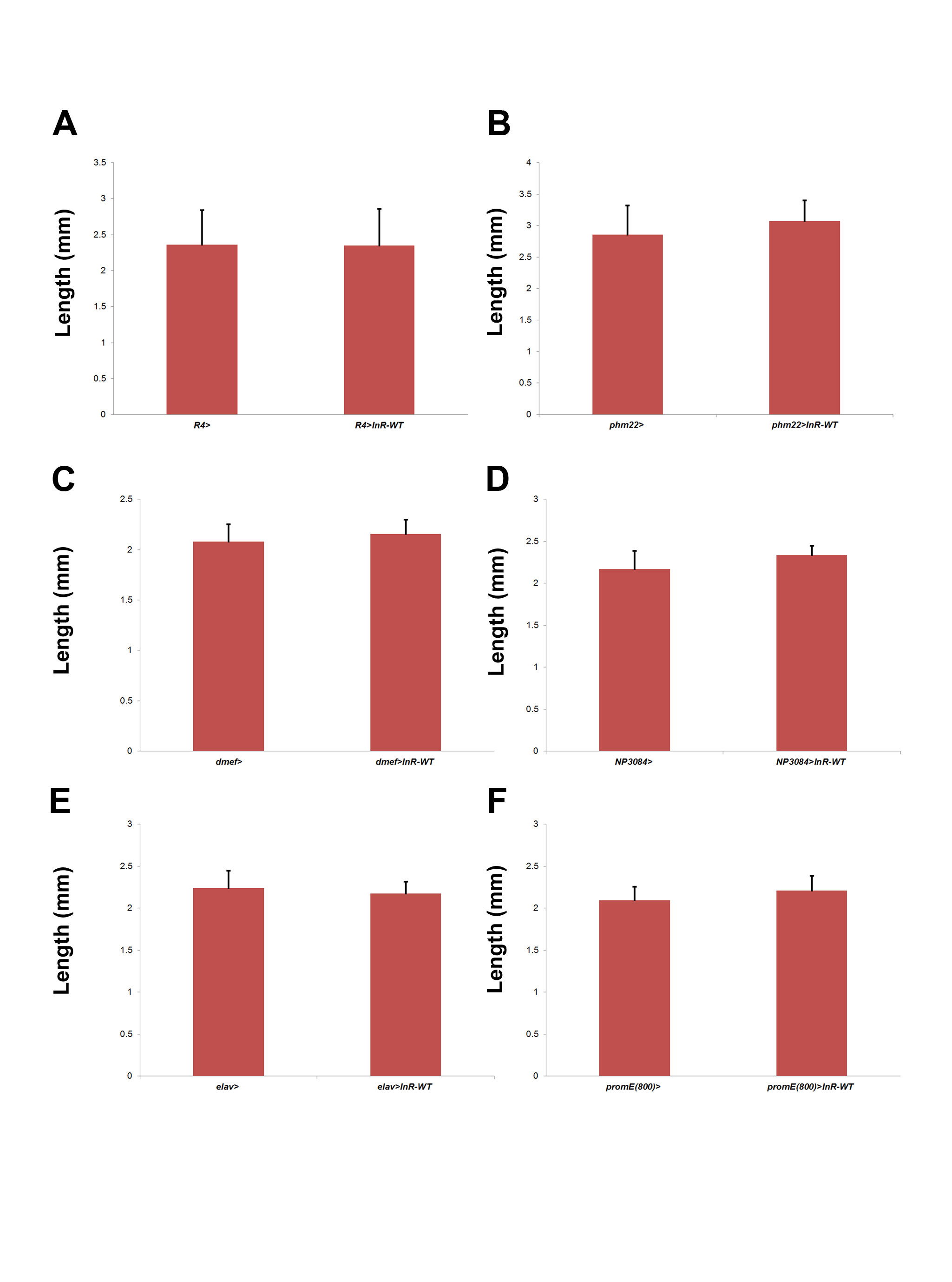

Supplement: S4 Fig — Survey of tissue-specific overexpression of the insulin receptor during hypoxia. [A–F] Under hypoxic conditions, overexpression of the wildtype insulin receptor in the fat body (R4>) (A), prothoracic gland (phm22>) (B), muscle (dmef>) (C), gut (NP3084>) (D), brain (elav>) (E), and oenocytes (promE(800)>) (F) did not lead to robust increases in larval size. Larvae in A were reared in 2.5% O2 from 42–72 hAH. Calculation of larval volumes for each genotype also did not show statistically significant differences (data not shown). (TIF) [file pone.0115297.s004.tif]

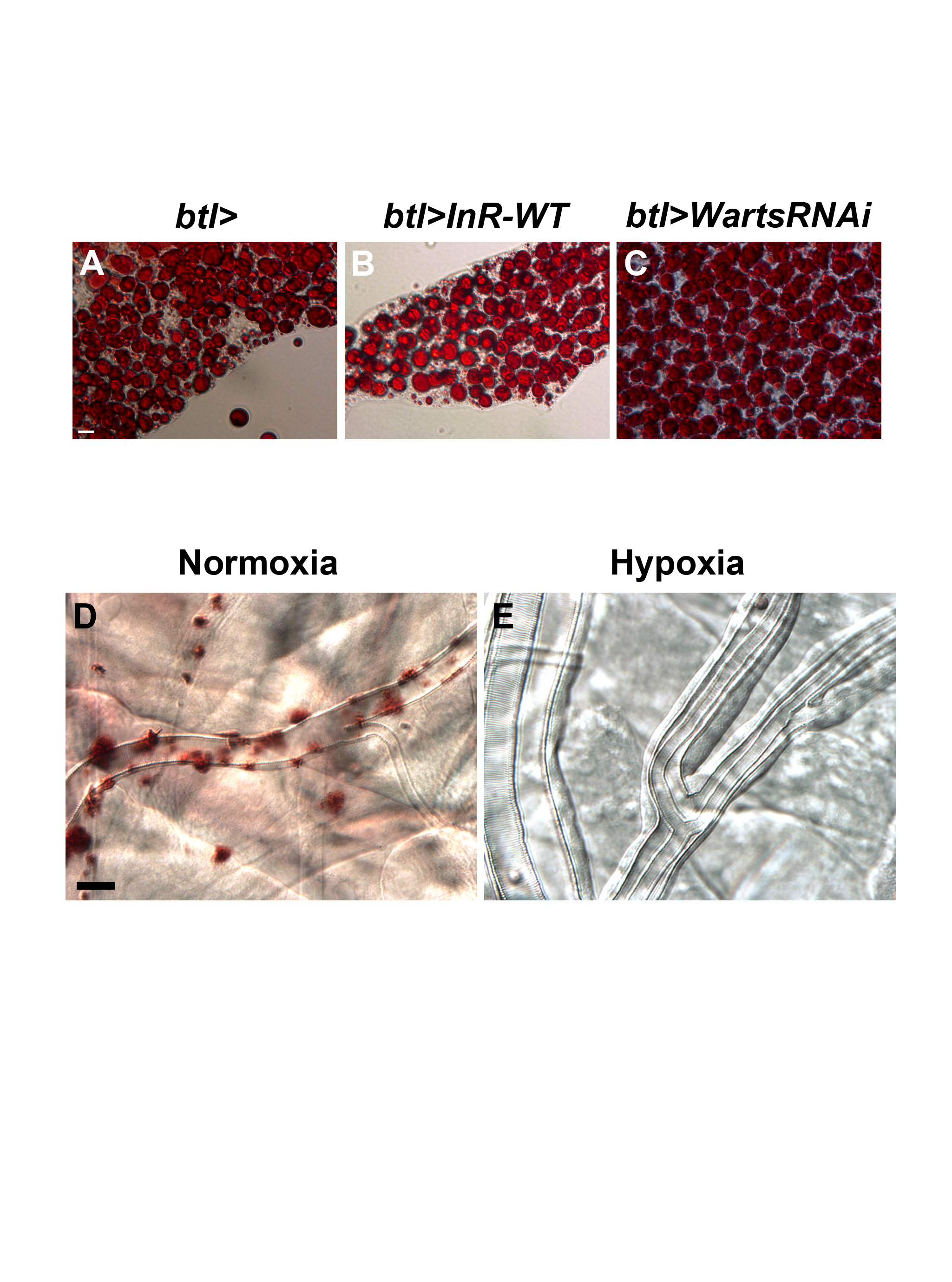

Supplement: S5 Fig — Lipid phenotypes in the fat body and trachea during hypoxia. [A–C] Overexpression of the insulin receptor (B) and downregulation of Warts (C) in the trachea did not reverse the hypoxia-induced lipid aggregation in the larval fat body, as determined by Oil-Red-O staining (A). Scale bar in A applies to A–C: 20 µm. [D–E] Under normoxic conditions, wildtype larvae exhibited lipid studding of their tracheal system (D), as visualized by Oil-Red-O staining. Under hypoxic conditions, lipid studding of the trachea was eliminated (E). Scale bar in D applies to D–E: 20 µm. (TIF) [file pone.0115297.s005.tif]
